# An Evaluation of the Mental Health Referrals to the Dales Living Well Team in Derbyshire

**DOI:** 10.1192/bjo.2025.10521

**Published:** 2025-06-20

**Authors:** Adeel Rauf, Mark Broadhurst

**Affiliations:** Derbyshire Healthcare NHS Foundation Trust, Chesterfield, United Kingdom

## Abstract

**Aims:** To examine the referrals in a newly established Living Well service at Dales community mental health team, Derbsyhire.

**Methods:** A retrospective review of notes on SystmOne (electronic patients records) referred for a period of 6 months from April–October 2024.

The source and rationale of the referral to the living well team, acceptance by the team, written communication to the referrer and the processes leading to the outcomes of the referral were examined.

**Results:** 75% of the referrals were from GP, 9% were from community IAPT teams.

67% of the referrals were accepted for input by living well teams.

70% of the referrals had a written letter sent to the referrer.

96% of the referrals led to a triage-based MDT meeting.

96% of accepted referrals had allocated member of staff making contact with the patient. 71% received a welcome call.

18% of the referrals had an outcome of being referred to the outpatients clinic.(as a long-term offer), 10% had psychology input as an outcome.

10% of the referrals were deemed after MDT discussion not to be appropriate for the service.

8% didn’t engage leading to discharge from the living well team.

15% of the referrals were due to symptoms of low mood, 14% with symptoms of anxiety, 12.2% of the referrals related to emotional dysregulation.

**Conclusion:** Two thirds (67%) of the referrals were accepted for a short-term offer by the team in providing support indicating a role of the living well team to provide prompt interventions regarding the mental health of patients.

A multidisciplinary approach in the team consisting of varied professionals has helped manage a lot of referrals with community-based support. 96% of the referrals were discussed with MDT approach.

A high proportion (70%) of the referrals had a letter written to the referral independent of the outcome. Written communication to the referrer is to be improved upon regardless of outcome of the referral.

A further qualitative review of the process to take place in 12 months time.

